# Children’s Hats

**Published:** 1880-12

**Authors:** 


### CHILDRENS' HATS.

Now that the sun is again occasionally visible, it may be worth while to remind parents that the use of a child's hat is to cover its head, and that the use of the brim is to shade the eyes. It is painful to see infants and little folks of tender years with half-closed eyelids, corrugated brows, and faces screwed up and distorted by the glare of the sunshine, from which they ought to be protected. Fashion is the Juggernaught of life all the world over, and children are tortured with the kindest intentions in the worship of the hideous monster ; but it is needless to inflict petty sorrows and annoyances which do not actually form part of the orthodox sacrifice to folly. While children are beneficently allowed to wear hats with brims, these useful appendages should be turned down so as to shade the eyes. This simple precaution will save considerable pain, spare some trouble with the eyes, and produce a more pleasing expression. Children who are perpetually struggling to keep the sun out of their eyes, do not either feel amiable or look happy, as a walk in one of the parks any fine morning must convince the attentive observer.—*The Lancet.*

